# Word Frequency and the Attentional Blink: The Effects of Target Difficulty on Retrieval and Consolidation Processes

**DOI:** 10.1371/journal.pone.0073415

**Published:** 2013-09-03

**Authors:** Stefan M. Wierda, Niels A. Taatgen, Hedderik van Rijn, Sander Martens

**Affiliations:** 1 Neuroimaging Center, University of Groningen, Groningen, The Netherlands; 2 Department of Neuroscience, University Medical Center Groningen, Groningen, The Netherlands; 3 Department of Artificial Intelligence, University of Groningen, Groningen, The Netherlands; 4 Department of Psychology, University of Groningen, Groningen, The Netherlands; University of Melbourne, Australia

## Abstract

**Background:**

When a second target (T2) is presented in close succession of a first target (T1) within a stream of non-targets, people often fail to detect T2–a deficit known as the attentional blink (AB). Two types of theories can be distinguished that have tried to account for this phenomenon. Whereas attentional-control theories suggest that protection of consolidation processes induces the AB, limited-resource theories claim that the AB is caused by a lack of resources. According to the latter type of theories, increasing difficulty of one or both targets should increase the magnitude of the AB. Similarly, attentional-control theories predict that a difficult T1 increases the AB due to prolonged processing. However, the prediction for T2 is not as straightforward. Prolonged processing of T2 could cause conflicts and increase the AB. However, if consolidation of T2 is postponed without loss of identity, the AB might be attenuated.

**Methodology/Principal Findings:**

Participants performed an AB task that consisted of a stream of distractor non-words and two target words. Difficulty of T1 and T2 was manipulated by varying word-frequency. Overall performance for high-frequency words was better than for low-frequency words. When T1 was highly frequent, the AB was reduced. The opposite effect was found for T2. When T2 was highly frequent, performance during the AB period was relatively worse than for a low-frequency T2. A threaded-cognition model of the AB was presented that simulated the observed pattern of behavior by taking changes in the time-course of retrieval and consolidation processes into account. Our results were replicated in a subsequent ERP study.

**Conclusions/Significance:**

The finding that a difficult low-frequency T2 reduces the magnitude of the AB is at odds with limited-resource accounts of the AB. However, it was successfully accounted for by the threaded-cognition model, thus providing an explanation in terms of attentional control.

## Introduction

It is well known that the human mind is limited in the conscious processing of relevant stimuli (*e.g.*, letters) when presented in close temporal proximity in a sequential stream of irrelevant stimuli (*e.g.*, digits). Most people show a reduced ability to successfully report a second target (T2) when presented within 200–500 ms of a first (T1), a phenomenon known as the attentional blink (AB) [1], [2]. Although there are a diversity of models and theories of this phenomenon, they can roughly be divided in two types: limited-resource accounts [3]–[5] and attentional-control accounts [6]–[9].

In limited-resource accounts of the AB, the common assumption is that there is a pool of resources available for processing targets and that this pool is limited. Whenever a target must be stored for later report, resources are drawn from the resource-pool in order to consolidate that target. Because this pool of resources is limited, there is a chance that the pool is still depleted due to the ongoing consolidation of T1 at the moment that T2 is encountered. Because there are not enough resources available for the processing of T2, an AB occurs. Thus, in these theories, a capacity-limitation of the attentional system underlies the phenomenon of the attentional blink.

On the other hand, there are theories that advocate an attentional-control account of the AB. The common theme in these theories is that processing of T1 is being protected by an attentional-control mechanism. Whenever a distractor is encountered, some kind of protection mechanism is trigged, preventing incoming information to be consolidated into working memory, effectively protecting the consolidation of T1. Because T2 is presented while T1 is being consolidated, the protection mechanism prevents T2 from being consolidated. Thus, whereas limited-resource accounts contribute the AB to a limited pool of resources, attentional-control accounts attribute the AB to some process actively suppressing the consolidation of new information.

In attempts to test and contrast these theories, several studies have been conducted that manipulated the difficulty of T1. Two types of difficulty manipulations can be distinguished: data-limited and resource-limited manipulations. Following the definitions of Norman and Bobrow [10], data-limited manipulations affect the physical characteristics of the stimuli (*e.g.*, contrast), whereas resource-limited manipulations affect the difficulty of a task (*e.g.*, number of candidate targets). Most AB studies that varied T1 difficulty employed data-limited manipulations, but the results have been mixed, with some studies finding an increased AB [11], an attenuated AB [1], [3], [12], or no effect [13], [14]. Others have reported data-driven difficulty effects on the AB, but only when T1 was not masked [15], [16]. It is known that if the distractors following T1 are replaced by blanks, essentially removing the mask on T1, the AB is clearly attenuated; the longer the duration of the blank interval, the smaller the AB [1], [3]. However, in the majority of AB studies, targets are typically masked by a subsequent distractor, which is often considered as a requirement to induce an AB (but see [17]). The mixed results by the studies described here make it hard to find conclusive evidence for either limited-resource or control-process accounts of the AB.

Although fewer in number, studies employing resource-limited manipulations show a more consistent pattern of results. Tasks that increased the informational load associated with T1 encoding typically produced a larger AB. Shapiro and colleagues [5] first showed that increasing the set-size from which a T1 could be drawn from 3 to 25 increased the AB. However, it should be noted that this was tested between rather than within subjects (10 in each group), and that data from the difficult condition (set size 25) was obtained from a different study (experiment 2 from [1]) using a slightly different procedure. An alternative explanation, for instance in terms of individual differences between groups, can thus not be ruled out.

In another study that manipulated difficulty to affect the AB [18], a T1 was used that consisted of five digits. The digits 0–4 were presented either in ordered (*i.e.*, ‘01234’) or shuffled (*e.g.*, ‘04231’) sequence. Participants had to report whether and in what sequence the target item occurred in the RSVP. The task for T2 was to identify a single digit represented by a 5-digit number (*e.g.*, ‘33333’). It was found that the ordered sequence produced a minimal AB compared to the AB produced by the shuffled digits. However, one should be cautious interpreting these results, for the ordered (low load) task could be seen as a recognition task (merely remember whether an ordered sequence was presented), whereas identification is required for the shuffled condition (report the full sequence). Furthermore, as the tasks for T1 and T2 were different, the effect of a task-switch potentially confounded the results [19], [20].

A third example of a resource-limited manipulation is provided by Martens and colleagues [21]. In their study, T1 difficulty was manipulated by changing the probability of occurrence associated with the identity of T1 (*i.e.*, one of the candidate targets occurred more often than the other target items). It was found that an infrequently reoccurring T1 target letter induced a larger AB magnitude than a frequently reoccurring T1 letter.

In a fourth study, difficulty of T1 was manipulated by varying the word frequency. Burt, Howard, and Falconer [22] showed that the AB is attenuated by word frequency. Participants had to identify two color-marked words in a stream of irrelevant pseudo-words. They found that high-frequency words induced a smaller blink than low-frequency words. The T2 word was always medium frequent. According to the authors, the T1 difficulty effects are more readily accounted for by limited-resource than by attentional-control theories.

Another line of evidence comes from event-related potential (ERP) studies. A late parietal component–the P300–has been associated with the AB [23]–[25], and is thought to reflect processes involved in the consolidation of targets into working memory [26], [27]. During the AB critical period, the P300 is suppressed for the second target [24]. However, earlier components associated to perceptual processing and the relatively late N400 (associated to semantic processing) can still be found [24], [28], [29]. These findings indicate that–to some extent–targets are being processed up to the semantic level, and are presumably accessed in memory, but are nevertheless not available for consciousness report. Therefore, the impairment seems to be at a post-perceptual stage of processing specifically related to the consolidation of a target for later report.

In addition, effects of the P300 found in AB studies could be taken as evidence in favor of resource-depletion theories, because manipulations that cause targets to elicit larger P300 amplitudes are generally found to increase AB magnitude, which suggest some kind of trade-off between the amount of processing and the probability that a target is detected [23]. For example, when a secondary task has to be performed next to the AB task, both P300 amplitude and AB magnitude decrease [30] (although resource-limited theories would have some issues explaining why a secondary task increases performance on the primary task). Indeed, some argue that the P300 can be used to index the allocation of resources [27], [31], [32], but one should be cautious to interpret the amplitude of the P300 as a direct index of resource allocation. For example, whereas high-frequency words are easier to detect than and induce a smaller AB than low-frequency words [22], they elicit a larger P300 amplitude than low-frequency words [33] (see [34] for a review of manipulation effects on the amplitude of the P300).

Several of the abovementioned studies have revealed evidence that the difficulty of the AB inducing task can influence the magnitude of the AB, but very few manipulated T1 difficulty within subjects without adding additional stimuli [21], [22]. As mentioned above, Burt et al [22] argued that their findings support limited-resource rather than attentional-control accounts of the AB. The goal of the current study was to replicate their findings and further investigate whether the AB is caused by a limitation in resources or by attention-control processes. Limited-resource accounts predict that performance should decrease when T2 is made more difficult. Because T1 and T2 are supposed to draw resources from the same limited-resource pool, the difficulty (in terms of frequency) of both T1 and T2 should affect the magnitude of the AB in a similar fashion. Predictions made by attentional-control accounts are more subtle. Whereas attentional-control theories also predict that a difficult T1 would increase the magnitude of the AB due to prolonged processing of T1, the predictions made for T2 are not as straightforward. Prolonged processing of T2 would affect the AB only at lag 2, when the protection mechanism for consolidation of T1 is trigged, and the effect could go both ways. Either the prolonged processing of T2 directly competes with both the processing and protection of T1, leaving no room for processing T2 and thus decreasing the probability of T2 to survive the AB period, or the prolonged processing could carry the target beyond the duration of the consolidation of T1 and its protection mechanism, increasing the probability of T2 to be consolidated. Thus, its prediction relies on the subtle timing of the target identification and consolidation processes.

Similar to the study of Burt et al [22], a natural manipulation was employed in the current study by using words that intrinsically varied in frequency of usage outside the context of the experiment. It is known that high-frequency words are processed faster and identified with greater accuracy than low-frequency words [35]. And because high-frequency words induce a smaller AB than low-frequency words, we assume high-frequency words to be easier targets than low-frequency words.

In the current study, targets consisted of words within a stream of unpronounceable non-words (Experiment 1) or within a stream of digits (Experiment 2). Target difficulty varied as a function of word frequency, without the need for stimulus degradation or other perceptual manipulations. Resource depletion theories predicted that overall identification performance for low-frequency targets would be lower than for high-frequency words, and more importantly, that a low-frequency T1 would induce a larger AB effect on a subsequent T2. Furthermore, a low-frequency T2 would require more attention or resources. According to most resource-limited theories, the largest AB was thus likely to occur for a low-frequency T2 following a low-frequency T1. Whereas attentional-control theories also predict a negative impact of a difficult T1 on the AB due to the prolonged duration of processes needed to identify and consolidate T1, the predictions made by attentional-control theories on the effect of difficulty of T2 are less straightforward. Difficulty affects the timing of different parallel processes, and as such can have either a positive or negative effect on the AB, depending on the onsets, offsets, and duration of cognitive processes during the critical AB period in which consolidation of T1 is being protected.

As described below, an attenuated AB was observed when T2 became more difficult, which is hard to explain with any resource-depletion theory. An extension of our threaded cognition model of the AB [8] is therefore presented, providing an explanation in terms of attentional control for this somewhat surprising finding. To confirm the finding that a low-frequency T2 is relatively easier to detect than a high-frequency T2 during the AB critical period, an ERP experiment (Experiment 2) was conducted in which only the word-frequency of T2 was manipulated. In line with the result of Experiment 1, and consistent with the model, again a relatively small AB was observed for low-frequency words when compared to high-frequency words. Also, smaller P300 amplitudes were found for low-frequency words when presented at long lags–consistent with findings that low-frequency words induce a smaller P300 than high-frequency words–but no difference between high-frequency and low-frequency words was observed during short lags.

## Experiment 1

### Methods

#### Participants

Twenty native German speaking psychology students (aged 18–25, mean = 20.4, with normal or corrected-to-normal visual acuity) from the University of Groningen were recruited via an online sign-up program, and received course credits for participating in the experiment. Informed consent was obtained prior to the experiment. The Ethical Committee Psychology of the University of Groningen approved the experiment.

#### Stimuli and apparatus

E-Prime 1.2 software was used to generate stimuli and to collect responses, running under Windows XP on a PC with a 17-inch 100-Hz CRT monitor. In total, 576 high-frequency (HF; Mannheim frequency 63 to 6413) and low-frequency (LF; Mannheim frequency 9 to 19) German words (four to six letters in length) were pseudo-randomly picked from the German word forms CELEX corpus [36]. The target words were balanced for word length and word frequency. Distractor stimuli were pseudo-randomly generated strings of consonants, consisting of the same number of characters as the targets on a given trial. The first letter of each word and non-word was presented in uppercase. The remaining letters were presented in lowercase. All stimuli were presented in black, Courier New font, size 18, on a white background at a viewing distance of ∼50 cm. The monitor’s resolution during the experiment was set at 1024×768 pixels.

#### Procedure

The experiment consisted of one practice block and three testing blocks, with a short break between each testing block. The practice block contained 9 trials and each testing block contained 288 trials.

The participants’ task was to identify two words (the targets) presented amongst a rapid serial visual presentation (RSVP) stream of non-words (the distractors). Participants were instructed to fixate on a cross in the middle of the screen. After pressing the spacebar, the fixation cross remained on the screen for 750 ms, followed by a blank screen. After 100 ms, the stream was presented, consisting of 22 stimuli. Each stimulus in the stream was presented for a duration of 150 ms without inter stimulus interval. T1 was always presented on the fifth temporal position within the stream. T2 was presented on the first, second, or seventh position after T1 (*i.e.*, lag 1, 2, or 7). Within each block, each combination of lag, T1, and T2 word frequency (HF-HF, LF-HF, HF-LF, and LF-LF), and word length (4 to 6) was presented equally often. A specific word was never presented twice on the same trial.

At the end of the stream, a question mark appeared, prompting participants to verbally report T1 and T2 to the experimenter. The correct answers were presented to the experimenter on a second display. Using the numeric keypad on a keyboard, the experimenter typed a “0” if a response matched with T1, a “1” if it matched with T2, a “2” if no response was given, and a “3” if it matched with neither of the targets. Responses were accepted and counted correct regardless of the order in which they were reported.

#### Data analysis

Following [30] and [37], accuracy scores were analyzed using binominal mixed effects models. Given that our hypothesis predicted a different number of observations per cell, mixed effects models are preferred over methods that assume an equal number of observations per cell. Analyses were performed using the lme4 package (version 0.999375-31) [38]. Lag, T1 word frequency, and T2 word frequency were entered as fixed factors in each model. For both word-frequency factors, the natural logarithm of the Mannheim word frequencies was entered in each model as continuous predictor. Subject was entered as random factor in each model.

### Results and Discussion

**Figure 1:**
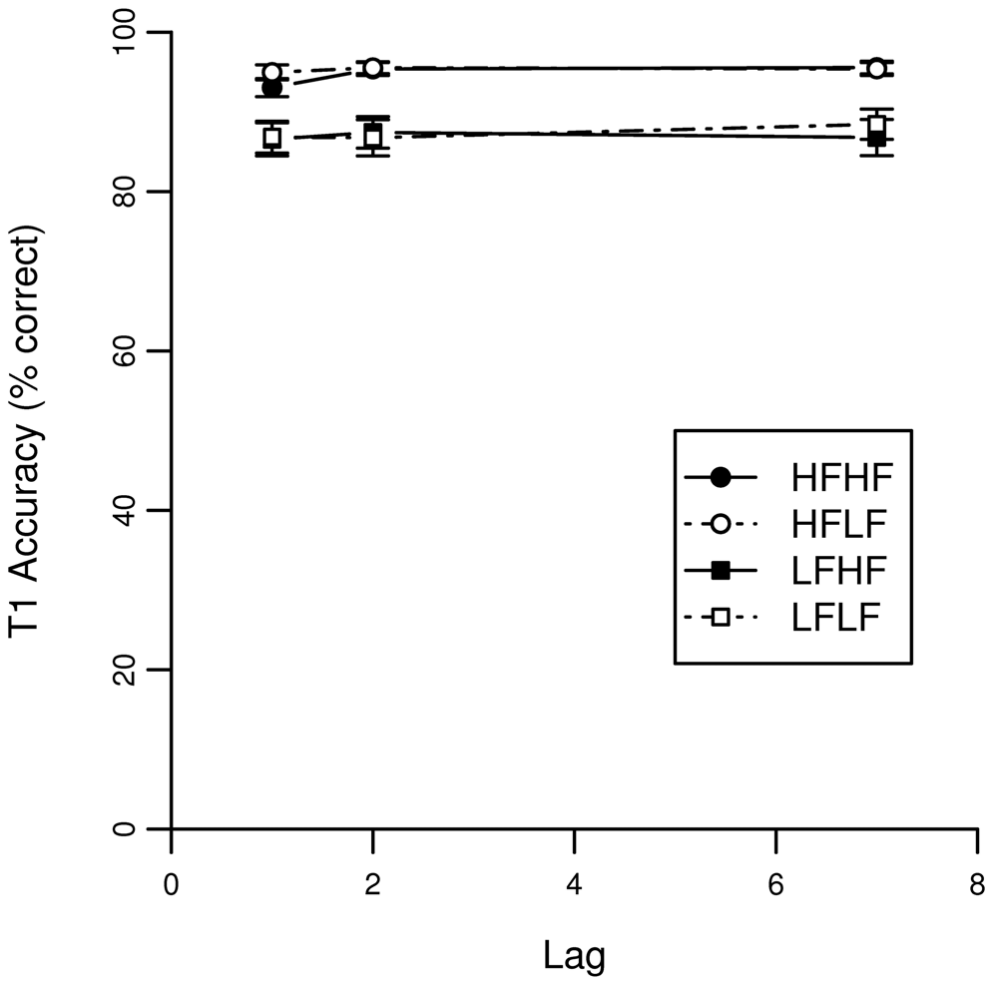
Accuracy scores of the AB task for T1 in Experiment 1. The lag corresponds to the temporal location of T2 relative to T1.

**Figure 2:**
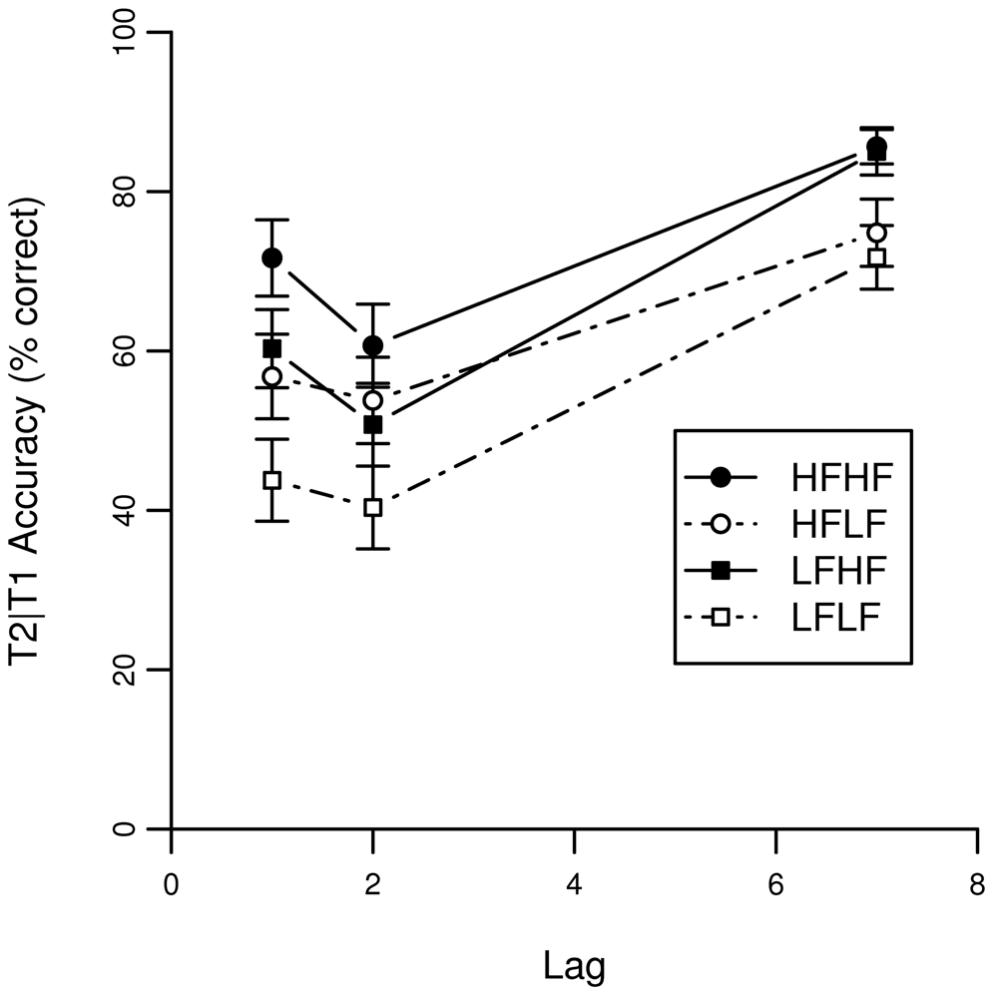
Accuracy scores of the AB task on lag 1, 2 and 7 for T2 given T1 correct in Experiment 1. The lag corresponds to the temporal location of T2 relative to T1.

Figure 1 shows identification performance of T1 as a function of lag. Identification performance of T2 given that T1 is correctly identified is shown in Figure 2.

#### T1 accuracy

A binominal mixed effects model was fitted on the accuracy of T1. Table 1 lists the statistics for the model’s factors. Here we will focus on the three significant estimates. The lag 7 condition was used as baseline and is reflected in the intercept. This factor indicates that a word with a natural logarithmic frequency of 0 would be responded to correctly in X% of all trials. The model revealed that T1 frequency predicts T1 accuracy, such that performance increases with T1 frequency (β = 0.308). The three-way interaction between Lag 1, T1 word frequency, and T2 word frequency (β = −0.063) indicates that if the two targets immediately follow each other, the positive influence of the word frequency of T1 on the accuracy is adjusted downwards as a function of the frequency of T2. This suggests that at short lags, the frequency of the second word might interfere with the processing of the first word.

**Table 1:** The estimates and z-values of the mixed-effects model for T1 accuracy.

	Mixed-effects model T1			
	Estimate β	Standard Error	z-value	p-value
(Intercept)	2.204	0.208	9.778	0.000
Word frequency T1	0.308	0.064	4.805	0.000
Word frequency T2	−0.054	0.048	−1.128	0.259
Lag 1	−0.188	0.178	−1.055	0.291
Lag 2	−0.288	0.180	−1.598	0.110
Word frequency T1, Word frequency T2	0.028	0.025	1.151	0.250
Word frequency T1, Lag 1	0.093	0.088	1.059	0.289
Word frequency T1, Lag 2	0.130	0.092	1.418	0.156
Word frequency T2, Lag 1	0.054	0.065	0.823	0.411
Word frequency T2, Lag 2	0.832	0.068	1.227	0.220
Word frequency T1, Word frequency T2, Lag 1	−0.063	0.032	−1.981	0.048
Word frequency T1, Word frequency T2, Lag 2	−0.039	0.034	−1.119	0.263

#### T2 accuracy

A binomial mixed effects model was fitted on T2 accuracy for trials with a correct T1 response. Table 2 shows statistics for each fixed factor. A marginally significant effect was found for the word frequency of T1, indicating that there is an overall long-lasting frequency effect (β = 0.073, p = 0.060) of T1. Interestingly, this marginally significant effect is positive, suggesting that a higher frequency for T1 is associated with better performance on T2. The main effect of T2 word frequency (β = 0.297) is similar to the effect of word frequency on T1 (β = 0.308), indicating that accuracy on T2 increases with higher natural-logarithmic word-frequencies in a similar manner as for T1. The negative estimate of lag 1 and lag 2 reflects the AB, showing that during the AB critical period performance is lower than outside the AB critical period (at lag 7). Furthermore, the interaction between T1 word frequency and both lag 1 and lag 2 demonstrates that the AB is modulated by T1 frequency. The positive estimate implies that the AB is larger when T1 is low frequent. Finally, an interaction between T2 frequency and lag 2 was found. The negative estimate indicates that the AB is relatively larger when T2 is highly frequent. This latter finding is somewhat surprising, as the effect is only found at lag 2, and one might expect a larger AB when T2 is difficult rather than easy, following limited-resource theories on the AB. However, in the next section, we describe a computational model of the AB that provides an explanation for this effect.

**Table 2:** The estimates and z-values of the mixed-effects model for T2|T1 accuracy.

	Mixed-effects model T2|T1			
	Estimate β	Standard Error	z-value	p-value
(Intercept)	0.880	0.260	3.381	0.001
Word frequency T1	0.073	0.039	1.878	0.060
Word frequency T2	0.297	0.044	6.687	0.001
Lag 1	−1.582	0.135	−11.679	0.001
Lag 2	−1.600	0.135	−11.840	0.001
Word frequency T1, Word frequency T2	−0.009	0.016	−0.532	0.595
Word frequency T1, Lag 1	0.162	0.051	3.196	0.001
Word frequency T1, Lag 2	0.144	0.051	2.850	0.004
Word frequency T2, Lag 1	0.017	0.056	0.299	0.765
Word frequency T2, Lag 2	−0.130	0.055	−2.348	0.019
Word frequency T1, Word frequency T2, Lag 1	−0.008	0.021	−0.379	0.704
Word frequency T1, Word frequency T2, Lag 2	−0.000	0.020	−0.011	0.990

### Model

**Figure 3:**
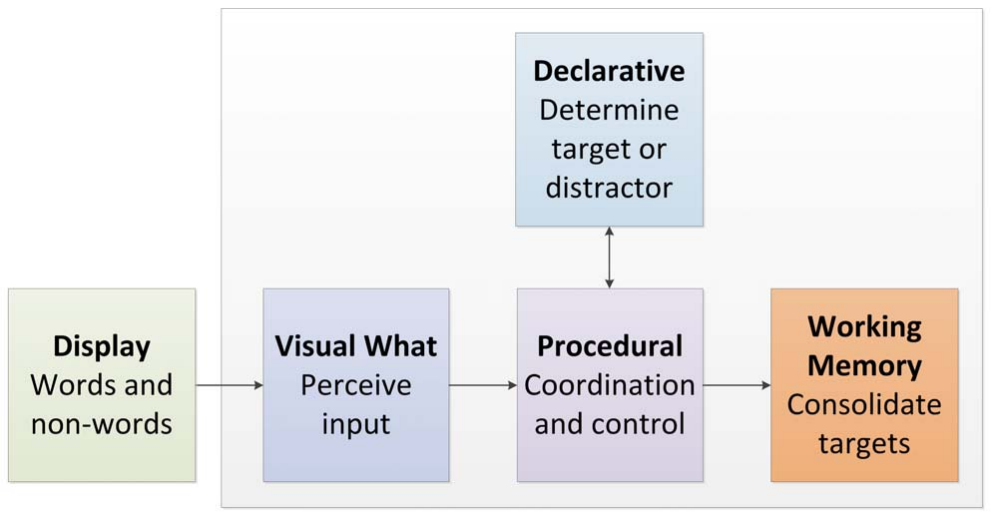
An overview of the modules and their role in the TC AB model.

In order to explain the patterns in the data, in particular the finding that a high-frequency T2 leads to a larger rather than smaller AB, we modified the threaded cognition (TC) model of the AB by Taatgen et al [8], to fit the current task. The TC model, which is based on the ACT-R cognitive architecture [39] assumes that several cognitive modules are involved in the AB task. More in particular, a visual module is needed to perceive the input, a declarative memory module is necessary to assess the category of an input (*e.g.*, target versus distractor), and an imaginal module is used to consolidate targets (comparable to working memory). Finally, procedural memory coordinates the flow of information (Figure 3). The TC assumption is that all modules can operate in parallel, but that a single module can only do one thing at a time. In the TC model, the AB is explained by a (procedural) control strategy that blocks the scanning for targets during memory consolidation. This control strategy is employed when a distractor is encountered. This explanation has similarities with those offered by some other models, in particular the Boost and Bounce model [7] and the eSTST model [9]. Specific about the TC model is that this control strategy has to compete with other processes, which enables it to explain why the AB is reduced in cases where there is distraction or a secondary task [8], [30], [40].

In order to fit the model to the current experiment, we changed the timing of the model to comply with the current experiment, and slightly changed the function of declarative memory. In the standard model, declarative memory was mainly used to determine the category of the stimulus, but now it is used to retrieve the representation of the word so that it can be reported later on. The assumption of the model is that the retrieval time of a low-frequency word is longer than that of a high-frequency word, and that the accuracy of identifying a word is also slightly lower. This is consistent with previous ACT-R models of lexical decision [35]. Furthermore, a second assumption is that it takes slightly longer to consolidate a low-frequency word in memory than a high-frequency word. Although an intervening distractor causes the AB in the model, performance on lag 1 (*i.e.*, no intervening distractor) is almost as low as performance on lag 2 (*i.e.*, during the AB period). Whereas the low performance on lag 2 is explained by the control strategy to protect T1 consolidation, performance on lag 1 is due to the direct competition between processes needed to consolidate T1 and T2. It is important to note that there was no difference in performance or fit between the modified model as presented in this study and the original model as reported by Taatgen et al [8].

**Figure 4:**
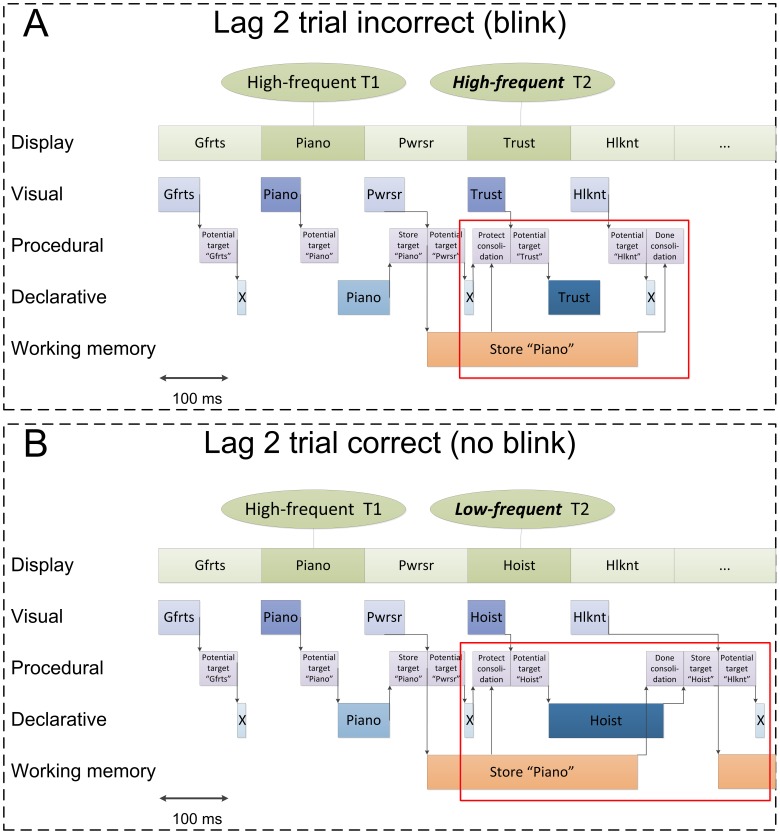
Examples of the model traces for the HFHF-condition (a) and the HFLF-condition (b). The second target was presented at lag 2.

**Figure 5:**
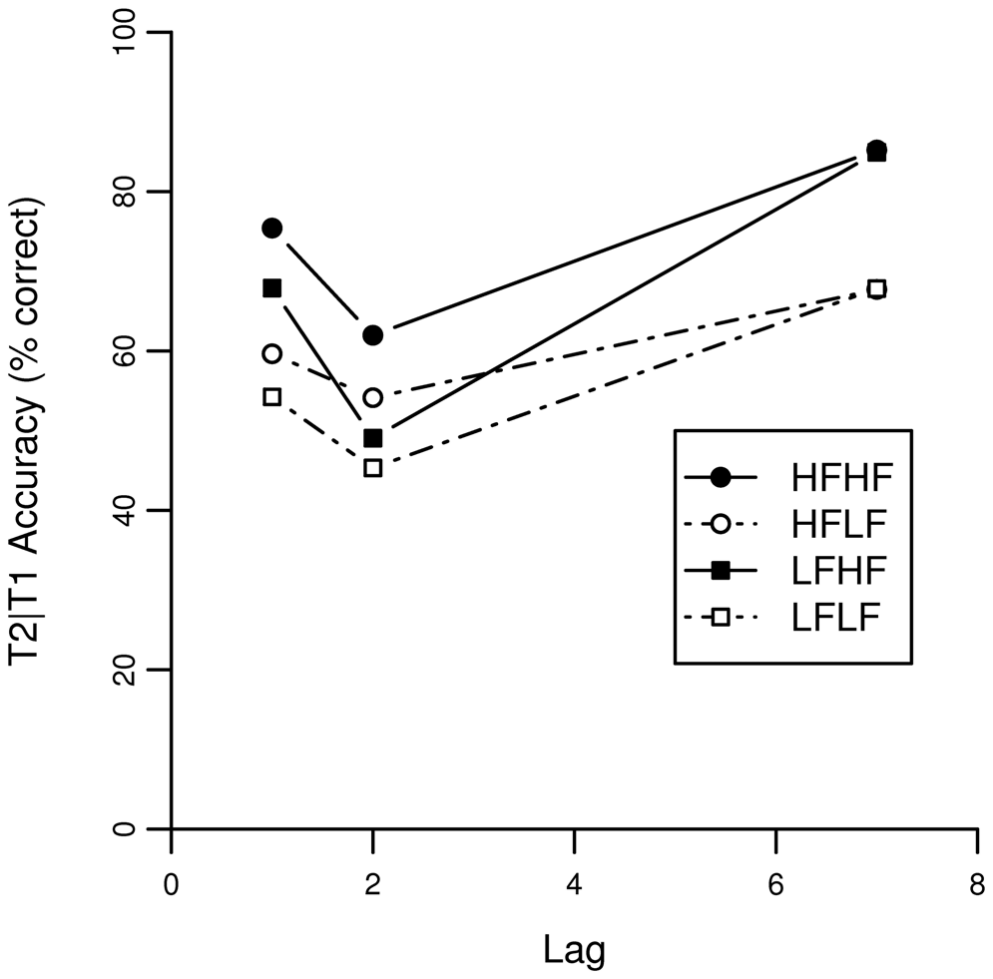
Accuracy scores of the AB task as produced by the model on lag 1, 2 and 7 for T2 given T1 correct. The lag corresponds to the temporal location of T2 relative to T1.

The crucial aspect of the model that can explain why the AB is relatively smaller in the cases where the T2 is of low frequency is that retrieving that word sometimes extends beyond the consolidation of T1, surpassing the strategic protection of consolidation. This is illustrated in Figure 4, where the activity of the four modules (along with a row representing the input) is displayed. Figure 4a illustrates a HF-HF trial in which there is an AB. After the word *piano* has been detected, the “Protect Consolidation” step in the procedural module temporarily prohibits targets from being consolidated, resulting in an AB. In the HF-LF example in Figure 4b, on the other hand, retrieval of the word *hoist* extends beyond the consolidation of T1, and therefore does not result in an AB. Because at Lag 1, no intervening distractor triggers the protection of T1 consolidation, the effect is absent for lag 1. The results of the model are shown in Figure 5, and fit the overall patterns in the data quite well.

In order to verify the results of Experiment 1 and test the hypothesis that indeed late processes were affected by word frequency, we set up an ERP experiment and focused on the P300 component, which is associated with late-stage processing of targets and is strongly related to the AB phenomenon (*e.g.*, [23], [24], [30], [41]–[43]). Providing converging evidence for the observed patterns of behavior, we expected to find ERP differences associated with late-stage processing of LF words relative to HF words. The amplitude of the P300 for a LF word was expected to be lower and the peak was expected to be later than those of a HF word. However, in line with our behavioral results and data from our computational model, these frequency-induced differences are expected to at least partially cancel out during the AB interval due to the fact that the late-stage processing of a LF T2 word extends beyond the period of T1 interference, escaping the AB more often than a HF T2.

## Experiment 2

### Methods

#### Participants

Twenty-one native German speaking psychology students (aged 19–24, mean = 21.0, with normal or corrected-to-normal visual acuity) from the University of Groningen were recruited via an online sign-up program, and received course credits for participating in the experiment. Informed consent was obtained prior to the experiment. The Ethical Committee Psychology of the University of Groningen approved the experiment.

#### Stimuli and apparatus

E-Prime 2.0 software was used to generate stimuli and to collect responses, running under Windows XP on a PC with a 17-inch 100-Hz CRT monitor. In total, 190 high-frequency (HF; Mannheim frequency 85 to 1,425), 380 medium-frequency (MF; Mannheim frequency 17 to 76), and 190 low-frequency (LF; Mannheim frequency 9 to 16) German words (four to eight letters in length) were pseudo-randomly picked from the German word forms CELEX corpus [36]. The first target word was always a MF word; the second target word was either a HF word or a LF word. Target-words were enclosed by ‘*X*’’s such that every stimulus had a length of twelve characters (*e.g.*, the word *BERGBAU* would be presented as *XXBERGBAUXXX*). Distractor stimuli were pseudo-randomly generated strings of digits, also consisting of twelve characters. Each word was presented in uppercase. All stimuli were presented in black, Courier New font, size 27, on a white background at a viewing distance of ∼50 cm. The monitor’s resolution during the experiment was set at 1,024×768 pixels.

#### Procedure

Similar to Experiment 1, the current experiment also consisted of one practice block and three testing blocks, with a short break between each testing block. The practice block contained 20 trials and each testing block contained 120 trials.

The participants’ task was to identify two words (the targets) presented amongst a rapid serial visual presentation (RSVP) stream of digit-strings (the distractors). Participants were instructed to fixate on a cross in the middle of the screen. After pressing the spacebar, the fixation cross remained on the screen for 500 ms. After the fixation cross disappeared, the stream was presented, consisting of 18 stimuli. Each stimulus in the stream was presented for a duration of 120 ms without inter stimulus interval. T1 was always presented on the fourth temporal position within the stream. T2 was presented on the second, seventh, or eighth position after T1 (*i.e.*, lag 2, 7, or 8). Within each block, each combination of lag and T2 word frequency (HF and LF) was presented equally often. A specific word was never presented twice in the experiment.

At the end of the stream, a question appeared, prompting participants to verbally report T1 and T2 to the experimenter. The correct answers were presented to the experimenter on a second display. Using the numeric keypad on a keyboard, the experimenter typed a “0” if a response matched with T1, a “1” if it matched with T2, a “2” if no response was given, and a “3” if it matched with neither of the targets. Responses were accepted and counted correct regardless of the order in which they were reported.

#### EEG recording

During the experiment, the EEG signal was recorded using a 64-channel electro-cap with tin electrodes (the organization of the electrode adhered to the international 10/20 system) connected to an REFA 8–64 average reference amplifier. Impedance was reduced to less than 10 kΩ for all electrodes. The data was sampled with a frequency of 2 kHz and digitally reduced to 500 Hz. The vertical electrooculogram (EOG) was measured from two tin electrodes placed approximately 3 cm below the left eye and 1 cm above the brow of the left eye. The horizontal EOG was recorded from tin electrodes attached approximately 2 cm to the outside corner of each eye. Two tin electrodes attached to the two mastoids served as an offline reference. Brain Vision Recorder (Brain Products GmbH, Munich, Germany) was used to control the data acquisition.

#### Data analysis

Preprocessing of the EEG data was done using Brain Vision Analyzer. Accuracy scores were analyzed using binominal mixed effects models. EEG data were analyzed using permutation tests and mixed effects models. Lag and T2 word frequency were entered as fixed factors in each mixed effects model. As in Experiment 1, the natural logarithm of the Mannheim word frequencies was entered in each model as continuous predictor. Subject was entered as random factor in each model. The *p*-values reported for the non-binominal models of the EEG data were calculated by performing 10000 Markov Chain Monte Carlo (MCMC) samplings. The permutation tests were used to determine the time-windows to be tested in the mixed effects models (as an alternative for visual inspection of the EEG grand-averages). Analyses were performed using the lmer and pvals.fnc functions in the lme4 (version 0.999375-31) [38] and languageR packages for the statistical software R.

### Behavioral Results and Discussion

**Figure 6:**
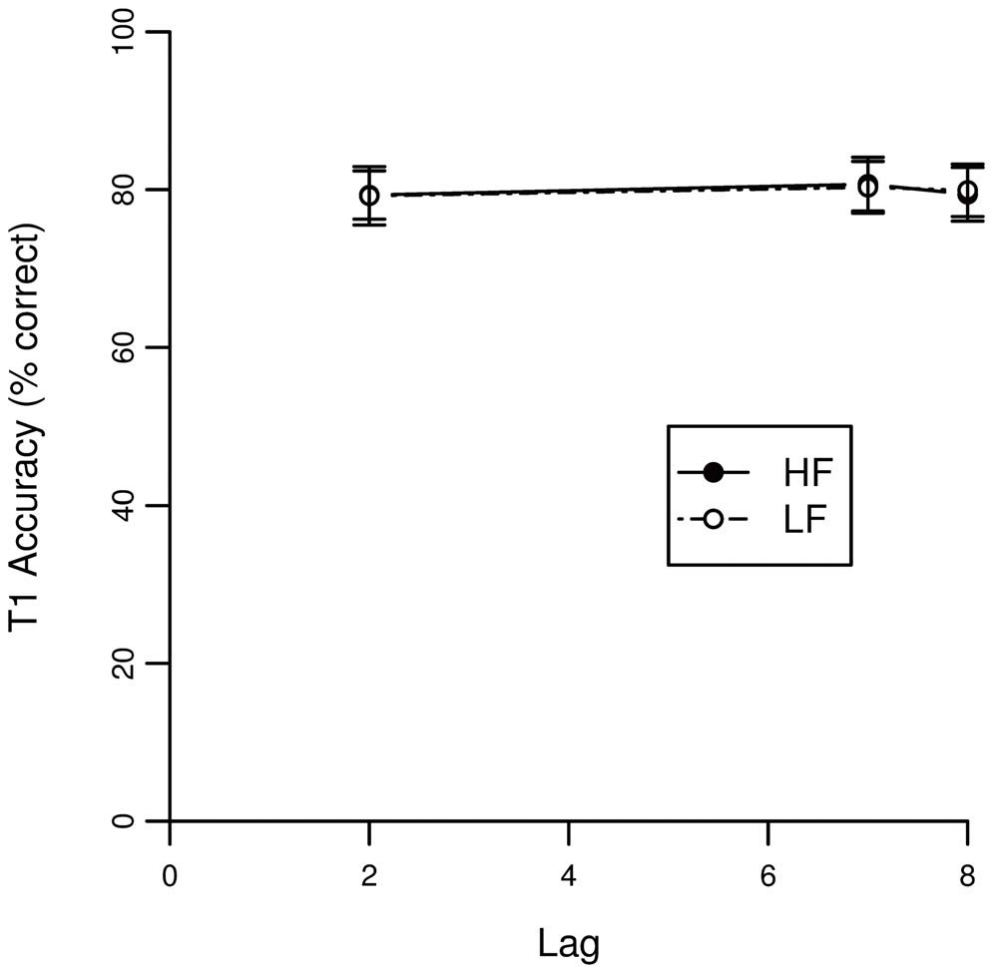
Accuracy scores of the AB task for T1 in Experiment 2. The lag corresponds to the temporal location of T2 relative to T1.

**Figure 7:**
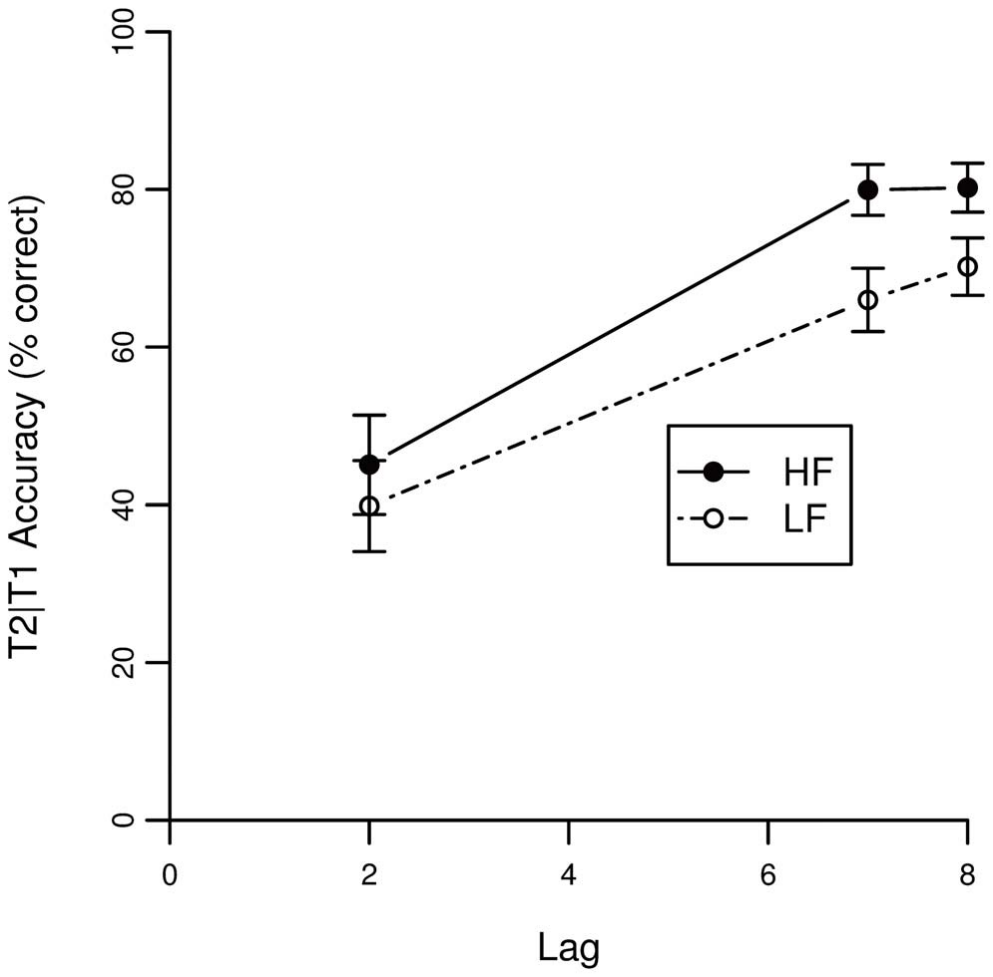
Accuracy scores of the AB task on lag 1, 2 and 7 for T2 given T1 correct in Experiment 2. The lag corresponds to the temporal location of T2 relative to T1.

Figure 6 shows accuracy of T1 as a function of lag. Performance of T2 given that T1 is correctly identified is depicted in Figure 7.

#### T1 accuracy

A binominal mixed effects model was fitted on the accuracy of T1. Table 3 lists the statistics for the model’s factors. None of the factors (*i.e.*, T2 word frequency and lag) significantly predicted T1 accuracy (note that T1 word frequency was not manipulated and thus not tested). These results are in accordance with the findings of Experiment 1 presented above.

**Table 3:** The estimates and z-values of the mixed-effects model for T1 accuracy.

	Mixed-effects model T1			
	Estimate β	StandardError	z-value	p-value
(Intercept)	1.501	0.252	5.968	0.001
Word frequency T2	0.009	0.039	0.226	0.821
Lag 2	0.044	0.227	0.196	0.845
Lag 7	0.117	0.230	0.510	0.610
Word frequency T2, Lag 2	−0.018	0.054	−0.335	0.738
Word frequency T2, Lag 7	−0.015	0.055	−0.277	0.782

#### T2 accuracy

A binomial mixed effects model was fitted on T2 accuracy for trials with a correct T1 response. Table 4 shows the statistics for each fixed factor. Again, a main effect of T2 word frequency is found (β = 0.226). The negative estimate of lag 2 (β = −1.027) again reflects the AB, showing that at the early lag performance is lower than at later lags. Confirming the results found in Experiment 1, an interaction between T2 frequency and lag 2 was found (β = −0.153). Again, the negative estimate indicates that the AB is relatively larger when T2 is highly frequent.

**Table 4:** The estimates and z-values of the mixed-effects model for T2|T1 accuracy.

	Mixed-effects model T2|T1			
	Estimate β	StandardError	z-value	p-value
(Intercept)	0.397	0.254	1.563	0.118
Word frequency T2	0.226	0.043	5.209	0.001
Lag 2	−1.027	0.226	−4.543	0.001
Lag 7	−0.322	0.238	−1.353	0.176
Word frequency T2, Lag 2	−0.153	0.056	−2.722	0.007
Word frequency T2, Lag 7	0.052	0.061	0.856	0.392

### Electrophysiological Results and Discussion

**Figure 8:**
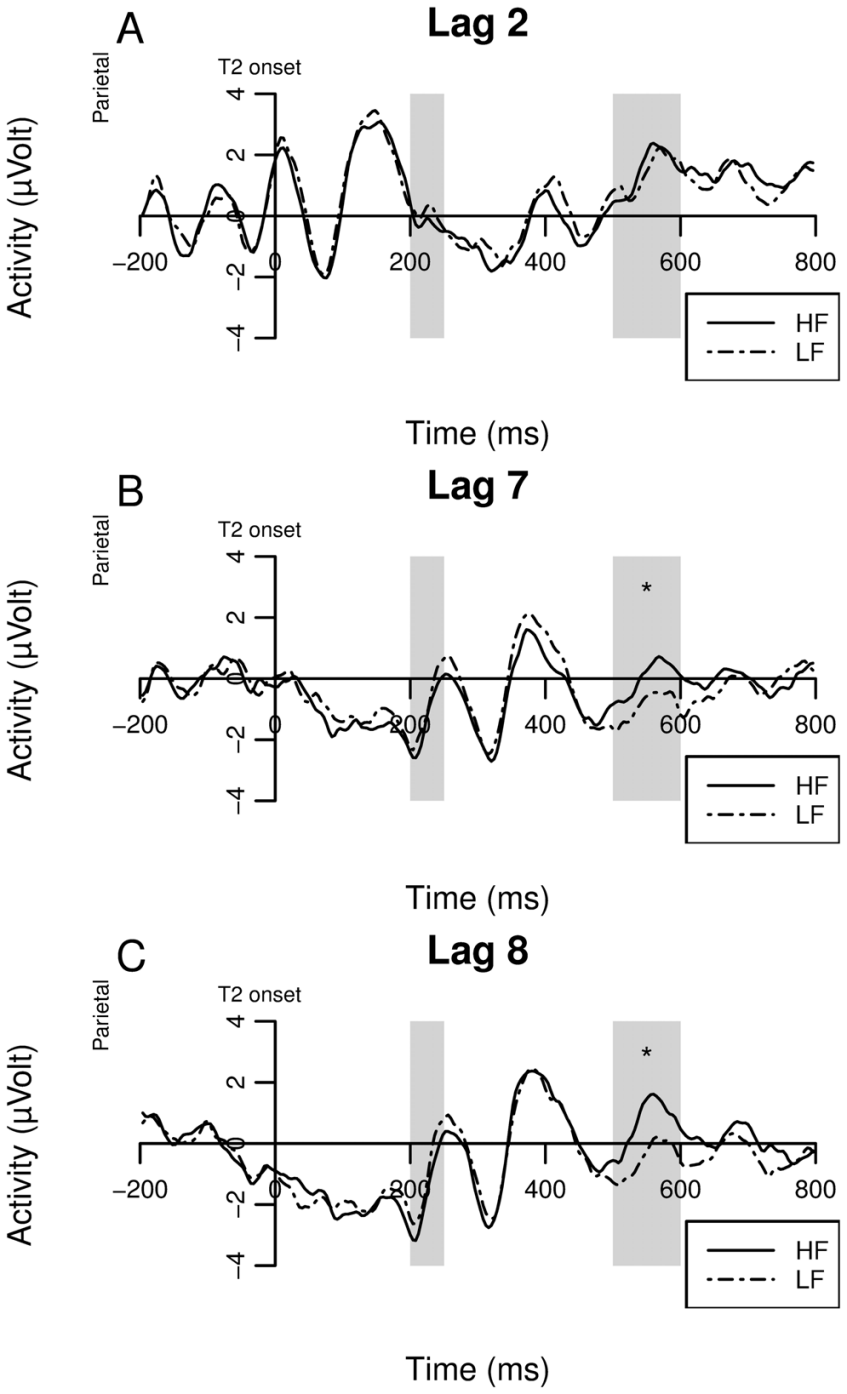
Grand averages for the ERPs of high-frequency and low-frequency words at lag 2 (A), 7 (B), and 8 (C). The ERPs are time-locked on target onset. Only correct trials were analyzed.

The EEG data was rereferenced to the mastoid electrodes. In order to remove noise, the data was filtered using a high-pass filter with a cutoff frequency of 1 Hz (24 dB/oct) and a low-pass filter with a cutoff frequency of 40 Hz (24 dB/oct). As we were interested in the activity at the parietal sites, data were then pooled over the parietal electrodes CPz, P1, P2, POz, and Pz. Next, the data were divided in T2-timelocked segments of one second (−200 ms to 800 ms). The 200 ms before onset of T2 served as baseline activity. Segments containing eye-blinks were excluded from analysis. Also, if the difference in voltage between the minimum and maximum data-point in a segments exceeded 100 µVolt, the segment was excluded. The final exclusion criterion was when the difference between two successive data-points exceeded 50 µVolt. In total, three segments were excluded. The grand averages for lag 2, 7, and 8 are shown in Figure 8a, Figure8b, Figure 8c, respectively.

#### Time-window determination

To avoid using visual inspection to determine the time-window of interest, permutation tests were used instead. For purpose of finding the window of interest, the data were binned in bins of 50 ms, resulting in a total of 20 bins. In the first permutation test, all lags where averaged together and the difference between high-frequency and low-frequency words were tested for each bin. To correct for multiple comparisons, the null-distribution was constructed from the maximum and minimum t-statistics across all bins of each permutation [44]. The null-distribution was constructed of 5000 randomly generated permutations. If the t-statistic was smaller or larger than the 0.025 or 0.975 quantile of the null-distribution, respectively, the bin was marked as a time-window of interest. In the first permutation test, a time-window from 200 ms to 250 ms (consisting of one bin, p<0.0446) and a time-window from 500 ms to 600 ms (consisting of two bins, p<0.0072 and p<0.0001) were found. The latter time-window was also found when the permutation test was performed on data from lag 7 (p = 0.0062 and p = 0.0068) and lag 8 (p<0.0230 and p<0.0020), but not at lag 2. The time-window from 200 ms to 250 ms was not found for the separate lags. A mixed-effect model for the first time-window did not reveal any effects of lag or word-frequency. Also, analyses on peak latencies did not reveal any evidence for latency shifts within the windows of interest (also, inspection of Figure 8 shows no indication of latency shifts within the windows of interest). The results of the mixed-effects model on amplitude differences for the time-window from 500 ms to 600 ms are discussed below.

#### Parietal late-positivity

As mentioned above, a mixed-effects model was used to analyze the mean activity in a time-window from 500 ms to 600 ms at the pooled parietal electrodes. We assumed that the late parietal activity is a late P300 component, as the time-course is similar to the P300 time-course found in literature [33] (note that the time-course is also similar to the P600 found in morphosyntactic-violation tasks, but this component seems to be distinct from the P300 [45]). The statistics of the model are shown in Table 5. The main effect of T2 frequency shows that parietal activity increases as word frequency increases (β = 0.388, p<0.001). Also, in comparison to lag 8, increased activity was found at lag 2 (β = 2.667, p<0.001). This is not surprising, as T1 related activity was likely to be present in the time-window at lag 2, but was absent in lags 7 and 8. Interestingly, an interaction effect of T2 frequency at lag 2 was found (β = −0.398, p = 0.016). Note that the size of the estimate is almost equal to that of the main effect of T2 frequency, but in the opposite direction (0.388 vs. −0.398), suggesting that the effect of word frequency was absent at lag 2. This indicates that the post-perceptual difference caused by word-frequency in successfully reported words disappears during the AB critical period.

**Table 5:** The estimates and z-values of the mixed-effects model for P300 amplitude (only correct trials are included).

	Mixed-effects model P300 amplitude			
	Estimate β	Standard Error	z-value	p-value
(Intercept)	−1.145	0.532	−2.152	0.031
Word frequency T2	0.388	0.099	3.937	0.001
Lag 2	2.666	0.701	3.804	0.001
Lag 7	−0.231	0.603	−0.383	0.702
Word frequency T2, Lag 2	−0.398	0.165	−2.418	0.016
Word frequency T2, Lag 7	−0.078	0.141	−0.554	0.580

Although we did not find the expected effects of latency, we did find amplitude differences in the P300. Outside the AB critical period, the low-frequency words were more likely to induce a relatively smaller P300 compared to high-frequency words. However, during the AB critical period, this difference was not observed. A likely explanation is that particularly words that were retrieved relatively quickly, mostly HF words that typically induce the largest P300 response, were more likely to be blinked. The net result is that the frequency-induced difference in P300 amplitude that was observed at late lags was absent at the early lag.

## General Discussion

Previous studies have shown that manipulating T1 difficulty can modulate the AB. However, so-called ‘data-driven manipulation’ studies in which the physical target properties (*e.g.*, contrast) were varied have often produced mixed results, or required T1 to remain unmasked. In addition, ‘resource-driven manipulation’ studies that changed the processing load rather than perceptual properties of T1 are both sparse and sometimes allow alternative explanations due to various methodological problems, including the presence of a task-switch, differing target-templates, or the use of small groups with between- rather than within-subject manipulations.

To address these issues, we manipulated the difficulty of both targets within subjects by presenting high- and low-frequency words as targets within a stream of distractor non-words. By virtue of the different frequencies that words have within a language, the difficulty of our word stimuli intrinsically varied in a more natural way than previous resource-driven difficulty manipulations. Based on findings from lexical decision studies (*e.g.*, [35]), low-frequency words were assumed to be more difficult than high-frequency words. In addition, it was predicted that a low-frequency T1 should induce a greater AB effect.

Consistent with the study of Burt et al [22], we found that a low-frequency T1 produced a larger AB than a high-frequency T1. Unexpectedly though, an easy high-frequency T2 produced a relatively larger AB than a more difficult low-frequency T2, when compared to performance at lag 7. This finding is at odds with limited-resource explanations of the AB, but can be accounted for in terms of attentional control and our computational model.

In our model, this relatively smaller AB observed for low-frequency T2 targets is attributed to the longer retrieval times of a low-frequency word from declarative memory. This is in line with findings by Polich and Donchin [33], who showed that the P300–an electrophysiological component associated with working memory consolidation–is delayed and its amplitude is decreased when a word has a low rather than high frequency. However, if the retrieval of T2 takes long enough so that it completes after the consolidation of T1 has completed, then T2 will be consolidated, reflected in a relatively smaller AB for low-frequency T2s. The combination of these orthogonal effects (a relatively small P300 for low-frequent words versus a relatively larger P300 due to a relatively smaller AB) may have led to the absence of significant word-frequency-related differences in P300 amplitude at lag 2. The explicit distinction in our model between the unconscious recognition of a target (the retrieval from declarative memory) and the conscious recognition of the target (the consolidation process) fits well with previous findings of post-perceptual semantic processing of blinked items (*e.g.*, [24], [46], [47]). Based on this explicit distinction, we predict that manipulations that shorten rather than lengthen the retrieval-time of a T2–by increasing its activation in declarative memory–might cause T2’s subsequent consolidation process to be blocked due to overlap with T1’s consolidation process, paradoxically leading to an increase in AB magnitude. For example, if one would conceptually prime a target T2 word by showing its pictorial counterpart beforehand, we predict that AB magnitude increases (for some initial evidence that this might indeed be the case, see [48]).

In summary, word frequency can be used to manipulate the difficulty of targets presented in RSVP in a resource-limited manner without introducing any sort of task-switching cost [20] or perceptual degradation. A low-frequency T1 word is more difficult to process and consequently increases the AB for T2, as reflected in the present results. However, the data indicate that if consolidation is delayed by a difficult T2–in our case through a prolonged retrieval from memory–there is a higher chance that the item will be successfully consolidated and reported. Simulations show that if the processing time prior to the consolidation stage increases, a T2 is indeed less likely to be blinked. The behavioral findings, computational model, and electrophysiological results presented here strongly support an attention-control rather than limited-resource account of the AB.
